# Proceedings of Societies

**Published:** 1859-09

**Authors:** 


### Proceedings of Societies.

NIAGARA FALLS, August 3d, 1859.

The delegates appointed by the various existing Dental Associations to meet at this time, with a view of forming a National Delegated Association, met according to previous notice. Dr. W. W. Allport was chosen chairman, and Dr. J. Taft, Secretary. On motion,

*Resolved,* That a Committee of three be appointed to examine and report on credentials.

*Committee.*—Drs. Taylor, Wright, and Suesserott.

REPORT OF THE COMMITTEE.

Upon examination the following persons were found to be duly authorized delegates, and present:—

*Mississippi Valley Dental Association.*—S. L. Hamlin, H. McCullum.

*Ohio Dental College Association.*—H. A. Smith, Jos. Richardson, J. T. Toland, J. Taft, G. W. Keely, Geo. Watt, E. Taylor.

*Pennsylvania College of Dental Surgeons.*—Wm. Calvert.

*Western Dental Association.*—D. W. Perkins, W. W. Allport.

*Penn. Association of Dental Surgeons.*—J. H. McQuillen, J. L. Suesserott, T. L. Buckingham, Geo. T. Barker.

*Indiana State Dental Association.*—J. F. Johnston, A. M. Moore, P. G. C. Hunt.

*St. Louis Dental Association.*—H. J. McKellops, I. Forbes.

*Pittsburgh Dental Association.*—W. M. Wright, R. Vandervort.

*Cincinnati Dental Association.*—C. H. James, J. G. Cameron.

*Ohio Dental College.*—James Taylor.

J. L. Suesserott,<br>W. M. Wright,<br>Jas. Taylor, } *Committee.*

On motion of Dr. McQuillen,

*Resolved*, That a Committee of three be appointed to draft a constitution, or plan of organization for this meeting. Committee—McQuillen, Richardson, W. M. Wright.

Adjourned till to-morrow, 4 P. M.

THURSDAY, 4 P. M.

The delegates met according to adjournment. The Committee on constitution for the purposed Association was called upon and reported. Before reading the constitution, Dr. McQuillen took occasion to make the following prefatory remarks:—

MR. CHAIRMAN AND GENTLEMEN:—The Committee appointed by this Convention to draft a Constitution, beg leave to state that they have attended to the duty assigned them; and would respectfully offer the following plan of organization. Trusting that its imperfections and short-comings may be remedied by *your reflections* and the *judgment* of *those* who shall participate in the final adoption of *a Constitution* for the proposed Association. A due regard for our own reputation as well as a just appreciation of the rights of others, make it obligatory that we should state, and desire the same to be placed upon record that, the plan of organization we submit to you is based upon a constitution that was framed by some of the brightest, and best minds of our Country, after years of deliberation. We refer to the American Medical Association.

After which the report was read.

On motion of Dr. James Taylor,

*Resolved,* That the report be referred back to the committee, with instructions to mature and amend, if necessary, and publish in the Journals as early as next November.

On motion,

*Resolved,* That this Association holds its next meeting in Washington City, on the last Tuesday in July, 1860, at 12 M.

On motion,

*Resolved,* That this Association suggest to the Dental practitioners residing in sections of the country, where there are no local or State societies, the propriety of forming such Associations, and electing delegates to meet this Convention at its next meeting.

On motion,

*Resolved,* That the delegates representing this Convention to be held next July, be practitioners of Dental Surgery, and that each College be entitled to one delegate, and each Association be represented by one delegate for each five members.

On motion,

*Resolved,* That Dr. James Taylor be appointed a committee to procure suitable rooms, and make all necessary arrangements for the next meeting.

On motion of Dr. Taft,

*Resolved*, That five persons be appointed by the chairman to prepare Essays to be read at the next meeting.

The following persons were appointed, each selecting his own subject.

Dr. J. TAFT. "Secretions of the mouth—Saliva and Mucus."

Dr. MCQUILLEN. ———

Dr. JOS. RICHARDSON. "The agencies concerned in the removal of the roots of the deciduous teeth."

Dr. GEO. WATT. ———

Dr. T. L. BUCKINGHAM. "Electricity—its connection with Dentistry."

Adjourned to meet at Washington City, D. C., on the last Tuesday of July, 1860. J. TAFT, Sec.

No other subjects than those designated for essays were selected for discussion. The subjects of the essays will be open for discussion, and the essays to criticism; this will probably occupy all the time that may be available outside of that employed for strictly business purposes.—ED.

---

### AMERICAN DENTAL CONVENTION.

### FIFTH ANNUAL MEETING.

BY OUR OWN REPORTER.

THE AMERICAN DENTAL CONVENTION met at NIAGARA FALLS, August 2d, 1859. The President, Dr. Isaiah Forbes, in the chair, and the other officers in their places.

The President, on taking the chair, welcomed the brethren to Niagara, and expressed a hope that their thoughts and actions, in the proceedings about to be conducted, would be as energetic, though less noisy than the great cataract itself. He hoped that, in any discussions which might follow, each member would state his own *peculiar* views, and omit every thing that was merely common-place or uninteresting. He stated farther that, to be entitled to the privileges of membership, it was necessary to enroll their names on the Treasurer's book, and pay to him the assessment levied by the Executive Committee, which, he stated, was two dollars for each member.

The minutes of the last day of the former session were read and approved. The President stated that, as the Report of the Executive Committee had been published, it would be regarded as adopted, unless objections were offered. The chairman of said committee, Dr. Allport, requested the privilege of changing the report, so as to admit of "Miscellaneous Business" at any stage of the proceedings in which it might seem proper to do so. The request was granted, by common consent.

The Treasurer, Dr. W. B. Roberts, presented his Report, which was accepted, and referred to the Executive Committee.

The committee appointed to memorialize Congress on the appointment of dentists to the army and navy, requested farther time, which was granted. Dr. James Taylor, of Cincinnati, is chairman of the committee.

A short recess was now announced, to give opportunity to subscribe the roll of membership, and pay the annual assessment.

The following is the list of members present, as enrolled on the Treasurer's book :—

*Maine.*—J. Mason, Saco; L. B. Straw, Bangor.

*Vermont.*—J. R. Lewis, Burlington; M. Tefft, West Poultney.

*Massachusetts.*—W. P. Dillingham, Boston; Abel Ball, Boston; W. D. Sanborn, Haverhill; J. Fisk, Clinton; D. B. Ingalls, Clinton; E. T. Wilson, Boston; S. P. Miller, Worcester.

*Connecticut.*—W. Potter, Norwich.

*New York.*—Geo. H. Perrine, F. H. Norton, T. H. Burras, Geo. Clay, E. A. L. Roberts, R. McIlroy, M. Levitt, James Pierson, C. S. Weeks, Sam'l Hassell, J. G. Ambler, B. W. Franklin, W. B. Roberts, New York City; L. W. Rogers,

Utica; L. F. Harvey, Buffalo; H. Hodge, Green; E. M. Skinner, Syracuse; Geo. F. Foote, Buffalo; H. A. Coe, Theresa; C. Elmendorf, Penn Yan; S. F. Tremaine, Rome; J. C. Gifford, Westfield; S. B. Palmer, Onondaga County; E. C. Hill, Niagara; E. Ware Sylvester, Lyons; T. Matson, Auburn; H. T. Tower, Lyons; A. Blake, Sardinia; Alfred Miner, Niagara; P. Harris, Skaneateles; P. B. Briston, Dansville; T. D. Evans, N. Y. Mills; D. S. Goldey, Oswego; A. Westcott, Syracuse; C. N. Whitney, Buffalo; S. W. Robinson, Watertown; John Lewis, Buffalo; E. D. Fuller, Peekskill; R. G. Snow, Buffalo; B. S. Whitney, Buffalo; S. N. Smith, Auburn; C. B. Foster, Utica.

*New Jersey.*—R. V. Jenks, Patterson.

*Pennsylvania.* — A. M. Asay, Philadelphia; H. B. Blair, Columbus; B. M. Gildred, Harrisburg; J. R. McCurdy, Philadelphia; A. B. Robins, Meadville; W. H. Luce, O. S. Elliott, Erie; J. L. Suesserott, Chambersburg; T. L. Buckingham, Geo. T. Barker, J. H. McQuillen, Philadelphia.

*Delaware.*—Wm. G. A. Boneville, Dover.

*Virginia.* —E. G. Winchell, Wheeling; Jona G. Wayt, Richmond.

*Georgia.*—D. S. Chase, Augusta; B.B. Alfred, La Grange; F. Y. Clark, Savannah.

*Missouri.* — Edgar Taylor, Palmyra; Isaiah Forbes, H. J. McKellops, St. Louis.

*Kentucky.*—Daniel Dougherty, Bardstown; H. McCullum, Augusta; J. L. Nourse, Cloversport.

*Illinois.*—W. W. Allport, Chicago.

*Ohio.*—W. H. Atkinson, C. R. Butler, G. Langsdorff, B. Strickland, Cleveland; R. McDowell, Wooster; E. P. Crowell, Cleveland; C. Palmer, Warren; Geo. Watt, Xenia; J. C. Burroughs, Warren; M. M. Oldham, A. A. Blount, J. Ramsey, Springfield; G. W. Keely, Oxford; C. M. Kelsey, Mt. Vernon; G. L. Paine, Xenia; C. P. Bailey, Cuyahoga Falls; H. A. Smith, S. L. Hamlin, Jas. Taylor, J. Richardson, C. H. James, J. G. Cameron, J. Taft, Cincinnati.

*Mississippi.*—Geo. S. Cook, Milford.

*Michigan.*—Wm. Cahoon, Detroit; C. B. Porter, Ann Arbor.

*Florida.*—P. P. Lewis, Tallehassee.

*Brazil.*—Van Tuyle, Brazil.

*Canada West.*—C. S. Chittenden, Hamilton; A. C. Crysler, Clifton; J. L. Walter, St. Catharine's; E. V. N. Relyca, Bellville.

*Wisconsin.*—D. W. Perkins, Milwaukee; L. George, Kenasha.

*District of Columbia.*—M. Loomis, Washington.

*Indiana.*—G. A. Wells, Indianapolis; Geo. Lupton, Shelbyville.

Miscellaneous business being in order, the President announced that he had received, through Dr. J. R. McCurdy, a letter from the "College of Dentists" of England. The reading of the letter being earnestly called for, Dr. McCurdy, by request of the chair, read the same, which is as follows:—

*To the Chairman of the Meeting of the "American Dental Convention," to be held at Niagara Falls, on Tuesday the 2d August,* 1859.

Dear Sir:—We are instructed by the Council of the College of Dentists of England, to address you on the occasion of your important annual gathering at Niagara Falls, in the ensuing month of August.

A few months since, many of the Dentists of this country anticipated the pleasure of inviting their trans-atlantic brethren to a general professional gathering in London, in the year 1861, at which period it was understood a universal Industrial Exhibition, similar to that of 1851, would be held.

The hope of such an Exhibition at the period indicated, is however, dispelled, in consequence of the present disturbed state of the continent of Europe, which has not only paralyzed all industrial efforts, but must inevitably retard civilization in many neighboring countries.

You are, doubtless, aware that differences of opinion in the ranks of our profession in England have tended, very materially, to check that unity of action which is so essential to the attainment of perfect organization and success; but we trust these differences will be speedily removed, so that, when the time is opportune for another "World's Convention," the *unanimous* invitation of the Dentists of England may go forth to their brethren in America. In this hope the Council most cordially indulge, and it would give to the several members great satisfaction, to welcome their American brethren to the shores of Great Britain, and especially with a view to the holding of a meeting in London, of the "American Dental Convention." We have the honor to be

Dear Sir, Yours faithfully,

GEORGE WAITE, M. R. E. S., *President.*

SAMUEL LOE RYMER, } *Secretaries.*

ANTH. T. HOCKLEY, }

5 CAVENDISH SQUARE, London,
15th July, 1859.

The Convention returned a hearty and unanimous vote of thanks to the "College of Dentists," for the interest manifested in the welfare of the profession in America. On motion, Dr. McCurdy was requested to return an answer, in behalf of the Convention.

On motion of Dr. Taylor, it was resolved that speeches be limited to ten minutes each.

At this stage of the business, much time was thrown away in discussing various schemes for obtaining a report of the proceedings of the Convention. The whole matter was finally left with the Journals.

The Convention next proceeded to the election of officers, which resulted as follows:—

*President*, L. W. RODGERS, Utica, N. Y.
*Vice-President*, GEO. WATT, Xenia, O.
*Recording Secretary*, FRANK FULLER, Portsmouth, N. H.
*Corresponding Secretary*, P. P. LEWIS, Tallehassee, Fla.
*Treasurer*, D. S. Chase, Augusta, Ga.

*Executive Committee.*

- GEO. F. FOOTE, Buffalo, N. Y.

- JOS. RICHARDSON, Cincinnati, O.

- T. H. BARRAS, New York, N. Y.

- J. MASON, Saco, Me.

- S. W. ROBINSON, Watertown, N. Y.

SECOND DAY, WEDNESDAY, AUGUST 3rd.

Convention met, President Forbes in the chair. The minutes were read and approved. The Executive Committee reported that they found the Treasurer's account correct, and that it shows that the indebtedness of the Convention is $12 04.

The address of the retiring President being next in order, Dr. Forbes arose and said :—

GENTLEMEN :—In seeing so many of you present this morning, I conclude that the thunders of Niagara have awakened the energies of my professional brethren; and, when I find that so many of you have brought your ladies with you, I conclude that it has also aroused your gallantry.

He then announced, as his subject, "*The duties we, as professional men, owe to our patients.*" And the first duty, he said, is to instruct them in the principles of our science—to endeavor to make them as well acquainted with the duties and responsibilities of the profession as ourselves. If ladies were present he would address himself directly to them, and beg leave to enforce on their minds the great responsibility resting on them. He would call to their mind the little babe, thrown into convulsions by the tortures of first dentition, and would ask them if they did not regard it as among their first duties to call in the aid of the family dentist, when the little nursling first showed signs of suffering from this cause. They might, however, object that they have not confidence in their dentist. Well, in former times they might not, but the dentist of *to-day* is entitled to their confidence. He would say to mothers, let no daughter come to womanhood with defective teeth; and especially, if that daughter were about to marry, see to it that her teeth are all in good condition, and her mouth healthy. This, he said, was important both for her comfort, and on account of the impaired vitality which might be transmitted to offspring by the opposite course.

Parents, he said, must be taught to recognize and distinguish the different classes and grades of teeth. When the first permanent molars are decayed, how often the parents rest content, and when called to notice them, tell us they have not shed them yet. Mothers are not better posted up on the first molars than they were twenty years ago; but this state of things must not be continued.

Our patrons must be instructed, he said, to call on the dentist before the nerve of the tooth is exposed. At our last Convention a member stated that whenever he found a pulp exposed, he thought "Why the —— (Sampson?) didn't you come sooner?" He could heartily sympathize with that member. In conclusion, he alluded in glowing terms to the life and character of the lamented TOWNSEND, whom all acknowledge as the father and founder of this Convention, and hoped that all would endeavor to imitate his virtues and strive after the same degree of excellence.

The retiring President now appointed Drs. Taylor of Cincinnati and Westcott of Syracuse, to conduct the President elect to the chair.

Dr. TAYLOR, in a few words, introduced President Rodgers to the Convention, who, before taking his seat made the following remarks:—

GENTLEMEN:—It may not become me to dispute the wisdom of the choice you have made for President; yet a single glance over this Convention assures me that whatever considerations of ability or fitness may have determined your choice, it was not made with primary regard to professional standing or attainments. And, while I fear I may not equal your expectations, even as a presiding officer, I am yet profoundly grateful for such an expression of your confidence and esteem.

Gentlemen: I am proud of the American Dental Convention. I have attended every session since it was organized, and have always been highly gratified with the personal appearance, general intelligence, liberal views, and social habits of those who have been present. And I will venture to say that for courtesy and gentlemanly deportment in their debates, for sobriety and freedom from all excess in their seasons of festivity, these gatherings will bear a favorable comparison with general conventions of any profession, trade or calling in the country. Such has been their character in the past,—What shall it be in the future?

It seems almost impossible to organize an association, even for religious and moral purposes, that will not soon be rent asunder by dissensions. If we would avoid such a result we must shun the rock on which others have split. Let us continue to cherish a spirit of kindness, concession and fraternal regard. While we freely discuss all questions of general interest, let us carefully avoid whatever is merely personal and local. We must strictly adhere to the rule that as a body we will not assume to decide upon the merits of any theory, invention, or mode of practice, but leave every member free to pursue his own course, responsible only to his patients and the community in which he lives.

And, Gentlemen, we must be willing to sacrifice something of personal opinion, for the sake of harmony; and in all that pertains to the organization and government of this body, submit cheerfully to the will of the majority. Union and harmony, under *any* organization, are better than dissension and division. "United, we stand; but, divided, we fall."

By strictly observing the same general policy which has guided us in the past, this Convention will become a permanent and useful institution—an honor to the profession, and a model for other associations.

Thanking you once more for the honor you have done me, I assume the duties of the office, relying upon your coöperation to enable me to discharge them satisfactorily.

DISCUSSIONS.

I. "*When, and under what circumstances, should teeth be extracted, to secure a correct development of the permanent teeth?*"

Dr. Taylor read a lengthy essay on this subject, which will appear hereafter in the original department of the Register. Many seemed to think this paper covered the entire ground, and were disposed to pass to the next question.

Dr. AMBLER, of New York, spoke of the great importance of this question, and of the conflicting opinions held in regard to it.

Dr. WESTCOTT moved that the question be divided, and the motion carried; but as none of the speakers (not even the mover), seemed to pay much attention to the division, the proceedings will be better understood by leaving it out of consideration.

Dr. WESTCOTT inquired if it was *ever* proper to extract the child's teeth till nature indicates the time by the absorption of the root, or protrusion of the permanent tooth. He said he often felt obliged to extract them before this—often long before. Here he differed from Dr. Taylor, if he had correctly understood the import of the paper just read. He remarked that it was difficult to speak on the subject, unless specific cases were presented.

Dr. PERKINS gave a case in which the patient, not yet four years old, had the four lower incisors protruding behind the temporary teeth, the latter being still firm. He asked Dr. W. how this case should be treated.

Dr. WESTCOTT said he would extract the temporaries.

Dr. PERKINS reported another case where the permanent centrals were too broad for the space between the temporary laterals, the centrals being half protruded and held in an irregular position by the laterals: he inquired of Dr. W. would he extract the laterals.

Dr. WESTCOTT replied that he did not come as counsel.

Dr. PERKINS, being called to answer his own question, stated that he would extract them; and, in all cases, where the retention of the temporary organs would cause irregularity of the permanent ones, he would extract without regard to age. It was often necessary, finally, to remove a permanent tooth, from each side, to make room. He also reported a case in which the temporary canines occupy the place of the permanent laterals. The patient is aged 17. He would not extract the temporary organs in this case.

Dr. WHITNEY said he was very frequently called on to extract the temporary molars, and that he always refuses, unless ulceration has taken place.

Dr. J. ALLEN said he advised parents to have the temporary teeth retained as long as possible. We should not be too much concerned about a little irregularity, but should remember that the jaw is still expanding, and that the temporary molars occupy more space than the bicuspids. He would not prematurely remove the temporary teeth unless compelled by disease. He thought the permanent germ was often destroyed by premature extraction of the deciduous organ.

Dr. CORMACK said there was no sure indication as to the time of extracting to prevent irregularity. He spoke of a case in which the temporary laterals were extracted to make room for the permanent centrals,—the permanent laterals never appeared, and the centrals, though crowded at first, separated to the extent of a line and a half. A daughter of this man's niece has retained her temporary laterals, which stand firm and are filled with gold.

Dr. SANBORN remarked that the temporary laterals might remain firm, longer than would be advisable to let the permanent centrals remain in an abnormal position. If the laterals be extracted, the centrals come into line, but the laterals come alike irregular.

Dr. ASAY said that, in estimating the probable results of any case of irregularity, it was important to see the parents. If both parents had broad, well developed dental arches, we might rest content that all could be righted in due time. If the parents were unlike in this respect, observe which parent the child resembles in dental development.

Dr. STRAW said that when the first permanent molar decays early, it is generally worth but little, and should be removed to make room.

Dr. CLAY said he had usually left the temporary organs as long as possible, but was now doubtful about it being the proper course. He mentioned a case in which the temparary lateral was removed, at nine to eleven months, and the permanent teeth came all right.

Dr. Coe inquired if the temporary molars were more liable to decay than the incisors.

Dr. Atkinson remarked that, to get a good development, it was necessary to have a healthy organization. He objected to the phrase "contracted jaw." A want of expansion by development and growth was the proper idea. He would rather have a denture very irregular, with the roots of the teeth perfect, than to choke off the deposit of bone for process, by too early use of regulating apparatus. He alluded to the prominence of the upper front teeth and processes of children caused by sucking the thumb, or an equivalent body.

Dr. McQuillen said it was important first to consider how the fangs of the deciduous teeth are removed. He was not satisfied with any of the theories ordinarily advanced on this subject. He thought we must look to the law that governs the functions of nutrition, absorption and deposition. Atrophy and growth depend on the ratio of these to each other. When an organ is not needed, or not used, it becomes atrophied, as is witnessed in the shrivelled arms of Indian devotees. He believed the matter of the old fang was used in making the new.

Dr. Taft thought we ought to examine principles rather than isolated cases in which we have not a knowledge of all the circumstances. He would let the temporary teeth remain, if healthy and the permanent are not making their appearance. If uncontrolably diseased, he would remove them. He did not know how far the development of the permanent teeth might be influenced by the presence of the temporary. If the pulp of a temporary tooth was dead, but no irritation resulted from its presence, he would let it remain.

Dr. Miller thought that but little is known on this subject as yet.

Dr. Westcott said that, when the question was divided, he thought there was but little left of the second part, but was now of a different opinion. He said that the permanent teeth will be developed in place or out of place, in spite of the temporary organs. He referred to cases in which perfectly formed permanent teeth were found deeply imbedded in the jaws, having never made their appearance through the gum. He did not know what causes the absorption of the deciduous fangs. He agreed with Dr. McQuillen, that it was not direct pressure. The theory of a carneous body eliminating an acid to remove them, he regarded equally as gratuitous as the idea of a peculiar acid being secreted at the tip of the tongue, to destroy the front teeth by abrasion.

Dr. Roberts spoke of a case in which a cuspid appeared late in life, but became a perfect tooth, and assumed its proper place in the arch. He inquired whether the tooth had previously grown to its normal size, and lain dormant in the process, or had failed to grow till near the period of its eruption. No one answered his question.

II. *Mechanical appliances and forces to correct irregularities of the teeth.*

Before commencing the discussion of this subject, it was resolved that members be restricted to one speech each, till all who desire to do so had spoken.

Dr. Westcott exhibited models and appliances illustrating his successful treatment of a case, in which all the upper teeth shut inside of the lower. We cannot do justice to this case in this report; but Dr. W. promised to describe it at length for the Register, when we will endeavor to have it fully illustrated with engravings.

Dr. W. B. Roberts recommended a perfectly fitting vulcanite plate, left thick adjacent to teeth which it is desirable to move. Then, by sawing a small notch in the plate, wedges of soft wood may be inserted from time to time, to keep up the desired pressure. If an inclined plane on the lower teeth was required, he also made it of vulcanite.

Dr. Asay recommended willow as the best wood for wedges.

Dr. Buckingham uses a stamped silver plate for making inclined planes. To expand the jaw, he uses a plate, and spreads it as occasion requires. The plate keeps the clasps from irritating the gums.

Dr. Franklin stated that many of the dentists in New York had exclusively adopted the vulcanite material, in the construction of regulating appliances.

Dr. Asay remarked that appliances for regulating should be worn night and day, and should be fastened, so that there could be no danger of swallowing them.

Dr. Westcott (by request) stated that as to the proper age for regulating, he would never begin till all the teeth are shed, and would not be discouraged even if the patient were not very young. He had not been successful with plates, in expanding the jaw as just described by Dr. Buckingham, but had with a bent wire with clasps pressing against the bicuspids and molars. A thread should be attached to the appliance and tied around a tooth to avoid the risk of swallowing it. He objected to the use of elastic substances in making pressure.

Dr. Atkinson made an inquiry in regard to the propriety of elongating the cuspids, or other front teeth, when they were too short.

THIRD DAY, AUGUST 4TH.

III. *Under what circumstances, and in what manner should teeth be filed, to remove superficial decay, or preparatory to filling, to leave them the most secure from future decay?*

Dr. Taft said that filing, to remove superficial decay, was practicable only on certain parts of the tooth; for example, it was practicable on approximate surfaces, but not in the depressions of the molars or bicuspids. It was more frequently serviceable on the front, than on the back teeth; and to be successful, it was necessary that the decay be quite superficial, that it can all be removed. A very slight concavity or depression might be admissible, in favorable positions on well organized teeth; but if the teeth be soft the decay will be renewed. We are commonly told, he said, to leave a shoulder, in filing the approximate surfaces of the front teeth; but, if their texture be soft, this he considered bad practice. The surface should, in all such cases, be as plain as possible. Nor was filing all that was necessary. The surface should be stoned, buffed and burnished. Some teeth, he said, would decay, almost as certainly as the bone was exposed.

As to the manner of filing, he would avoid, as far as practicable, the mutilation of the front teeth. He would, therefore, let the space be greater posteriorly than anteriorly. If there is a tendency to inflammation, it is necessary to manipulate cautiously. He would support the teeth while filing. A cutting instrument, he said, was often preferable to the file. It produces less irritation, and inflicts less pain. It should, however, be followed by a fine file.

Dr. Atkinson said it was important to have a few basal facts, or principles, that all could understand. If the decay did not extend through the enamel, the skillful use of a fine file, followed by a proper polishing material, rendered the tooth as good as ever. If through the enamel, and consolidation of the dentine had taken place, he would pursue the same practice; but, if the dentine is softened, he would expect little or no benefit from filing.

Dr. Rogers inquired how far the enamel preserves the tooth, and suggested that polished toothbone would resist decay as well as enamel.

Dr. Chase thought that great care should be taken not to deform the teeth by the use of the file. If filed so as to fall together again, decay would be renewed on the filed surfaces. He would leave the spaces between the front teeth wider posteriorly than anteriorly. He would not have the filed surface a perfect plain—would make them oval on the molars, so that the tongue could displace particles of food; and, in general, he would leave a shoulder, to prevent the contact of the filed surfaces.

Dr. PERINE thought there was danger of using the file too much and too harshly; and said that the temperament of the patient should always be taken into consideration.

Dr. FULLER, of Peekskill, recommended oxyd of tin (tin putty) as a polishing material for filed surfaces. A free use of the file was generally, he thought, the most successful. On molars, he left the space v shaped, wider lingually than labially, so that the food would be pressed toward the cavity of the mouth. In front teeth he seldem left a shoulder, usually left the surface somewhat like the mould-board of a plow. Foreign matter would glide off the surface better than if a shoulder were left.

Dr. WESTCOTT said there were two specific questions on the subject under consideration. He would notice only the latter. Age must be considered. With children, he would wedge the teeth apart, and avoid filing approximate surfaces, if practicable. He would wedge, if the teeth were close together, so that he could file properly. In all delicate cases, he would pursue the same course; but, in *dirty* mouths, he would walk right in with the file, and file as long as he dared. As to the filed space between molars he would leave the wide end of the v outward or inward, as the position of the cavity might indicate. Sometimes he did not even run the file clear across the space between the teeth.

Dr. TAYLOR said he agreed mainly with the remarks of Drs. Fuller and Westcott. In that class of decay by which all the tooth substance is removed from the cavity, he would file, and also when the tooth, by its increased sensibility, showed a disposition to resist decay. He would file off, even the whole labial surface, if necessary; and then, if the mouth were kept healthy, the teeth *would* last. When a molar and bicuspid were decayed on their adjacent approximate surfaces, he would cut the decay from the one and file the other.

Dr. Forbes said that in superficial decay he does n't file, and never did. He told the patient that the tooth would soon need attention, and that he *must* have it attended to in due time, or it would be lost. He preferred to trust the patient's care, rather than the file. He remarked that, as early as 1820, it was fashionable to file front teeth, to separate them, for sake of appearance, and that such teeth usually decayed.

Dr. W. B. Roberts said that, like Dr. Forbes, he lets superficial decay alone till it is deep enough to fill. In filing teeth, he aimed to leave the tooth as nearly in its natural shape as possible. In separating teeth, he preferred wedging to filing, when practicable.

Dr. Weeks said that he once had strong prejudices against the file, but now uses it freely. He wedges, however, when practicable.

Dr. Allen said the file is a valuable instrument; but it must not be used indiscriminately. For superficial decay, he would file and burnish. He had seen filed surfaces remain sound for twenty years.

Dr. Vandevoort said that he found filing more successful in black decay, than in other varieties.

Dr. Wilson wished to know how Dr. Forbes gets along without the file, in superficial decay.

Dr. Forbes explained that he considered the tooth safer, by throwing the responsibility of its care on the patient, rather than by filing it, and giving the impression that it was thus rendered safe.

Dr. Perkins said that when the labial surface of a front tooth was decayed, by removing the decay with a file or excavator, and then polishing the surface, it would last, *at least as long* as if the decay were not removed. He tried the experiment with two teeth thus decayed to about the same extent. One he filed and polished, the other he let remain as it was. Three years after, the filed tooth was good, and the other required filling.

Dr. Butler would not file, expecting that decay would thereby be arrested, except on anterior, or other favorable surfaces. If a filed surface was kept clean, till consolidation takes place, he regarded the tooth as comparatively safe.

Dr. Ambler said it was not the proper, but the injudicious use of the file that gave rise to prejudices against it. His views on the file were directly opposed to those of Dr. Forbes.

[Some good, sharp jokes were passed between Dr. A. and Dr. F. on the subject, but as we do not report the fun, the reader must go and hear for himself next time. W.]

Dr. Solyman Brown, of New York, was heard at some length in regard to the Biographies of Dentists, which he proposes to publish.

On motion of Dr. John Allen, it was resolved that this Convention is glad to learn that Dr. Brown contemplates the publication of such a work.

Pending this subject, a motion was made which seemed to be unacceptable, and the mover asked leave to withdraw it, which was granted by unanimous consent. Immediately a member offered a resolution the same in substance as that withdrawn. The chair decided this to be not in order. The mover appealed, and the chair was heartily sustained.

IV. *Filling Teeth.*

Dr. Perine exhibited an instrument for compressing the sublingual ducts. The instrument is the invention of Dr. Hawes, was manufactured by J. D. Chevalier, and will, doubtless, be kept for sale at all the Dental Depots.

Dr. Franklin recommended a section of an India rubber ball, placed between the teeth and cheek, to compress the perotid duct. The outlet of the duct should be covered with blotting or bibulous paper, and the convex surface of the rubber section placed upon it.

Dr. Ambler spoke of a mode of fang filling, which he practiced when cheapness was necessary. He fills the apex of the fang with gold, the body of the canal with gutta percha, put a cap of gold over this, and fills the cavity of decay with gold.

Dr. Strickland stated that for a long time he had kept ready drawn, pure gold wire for filling fangs. He formed the wire according to the size of the instrument used in cleaning the canal, and then forced it up to the apex. More than twenty years ago he adopted the theory that, if the foramen was thoroughly closed, it mattered but little whether the canal was filled or not.

Dr. Forbes said that, in America, the servant girl *would have* as good work in her mouth as her mistress. He thought that to study how to do cheap work had a tendency to introduce bad work.

The work of filling he would consider under five divisions; 1st, The preparation of the cavity. 2d, The preparation of the gold. 3d, Preparing the mouth. 4th, Filling the tooth. 5th, Finishing.

1. He would take an instrument so large that it would not enter the cavity, and remove all superficial discoloration. He would break down the borders till the walls of the cavity were perpendicular. He would cut down to the bottom, with his favorite gouge instruments. Then he would bevel, or countersink, the mouth of the cavity, as if for a screw head.

2. Cut a book of No. 4 foil, book and all, fold into strips of eight thicknesses, make cylinders of these strips, by laying each strip on a cushion and rolling it on a broach.

3. The only difficulty in preparing the mouth is with the under teeth. He would surround the tooth with a piece of muslin, or a napkin, and support it with his fingers.

4. Suppose the cavity to be three lines in diameter, and the same in depth. He would take a cylinder, having the same diameter, but long enough to project a line out of the cavity when standing on its bottom. With a flat instrument, he would press this cylinder firmly to one side. The cavity would thus be about half filled. The remaining half he would fill part way up, as it would be difficult to get a slim cylinder to go entirely to the bottom. With short cylinders, he would fill the remainder of the cavity. With a pointed instrument he would pierce the surface of the plug, where practicable, and fill in with adhesive gold.

5. He would finish in the usual way.

Dr. CLARK, of Georgia, formerly used adhesive, but now prefers non-adhesive foil. In the South, on account of excessive perspiration, it was important that the fingers touch the foil as little as possible. He usually fills front teeth with strips, and the others mostly with cylinders. He exhibited a very convenient apparatus for folding foil into strips of any desired width. The apparatus consists of a pair of ivory rulers, beveled to a thin edge at one side, and left thick at the other. The foil is laid on a flat surface, one of the rulers is laid on it, and the thin edge of the other is passed under it. By passing the one toward the other ruler, the free part of the foil is folded over it. The process is repeated till the strip is as narrow as is desired.

Dr. ROBERTS reported a case of an upper central, with one corner broken away, which had been filled with amalgam. Inflammation and death of the pulp resulted. He removed the amalgam, cleaned out the canal, drilled through the apex, and injected the canal with solutions of nitrate of silver. When restored to health, he inserted a platinum wire into the canal, letting it project so as to support the gold used in building out the corner, to restore the shape of the tooth. The case is now doing well.

The order of business, by common consent, was now temporarily suspended, and Dr. Frank. Fuller moved that the Treasurer be instructed to pay to Dr. Potter $154 20, being the balance of his expenses in defending a suit for infringement of what this Convention regarded as a spurious patent. After some explanatory remarks, the motion was carried unanimously.

Dr. FORBES, resuming the discussion, said that in filling fangs, he used wire, feathered the end of it, wrapped it with foil, and drove it "home."

Dr. Frank Fuller, in filling crown cavities, preferred to have them cone shaped—wider at the mouth than at the bottom. Taking square pieces of gold, he would then press them down in the middle, leaving the edges projecting. When the cavity was full, he would condense firmly, first the edges, then the entire surface. He was opposed to under cuts.

Dr. Bristol said that this class of cavities was easily managed; but cases occur in which it is impossible to have the orifices larger than the rest of the cavity. The cavity must sometimes be "under cut," and he would inquire if there was no successful method of filling such cavities. Such teeth would not admit of such powerful compression; and he did not see how Dr. Forbes successfully condensed such large cylinders as he described.

Dr. Taylor said it must be understood that there will be exceptions to any rule. In some cases he preferred to have the cavity larger within; and it was sometimes best to fill the under cuts first, and then finish as in plain cavities. He would let the first block, or cylinder, lie against a straight wall. There was no difficulty in condensing cylinders laterally, as it did not require much force to press parallel layers of foil against each other. The first block introduced should be so large that it will stay in place, when condensed.

Dr. Barker said he agreed with the remarks made generally on this topic; but no difficult cases had been presented. None that a tyro could not do justice to. He would like to hear how difficult cases are to be treated, for example, spoon shaped cavities.

Dr. Atkinson said this was altogether too important a subject to be fooled with. Definite rules were needed, and he would address himself to those who were now in the situation he once was. The most difficult case is where there is a strong predisposition to periosteal inflammation, as the tendency of this is to render the tooth a foreign substance. If the patient could bear the operation, he would clean out the canal, and fill to the apex. The canal might be cleansed, and the tooth filled the same day, unless it was desirable to bleach it. As a dressing for the canal he would use a thread, strong enough not to break, applying alternately, creosote and tincture of iodine.

He had been requested to give his views of the use of the mallet in filling teeth. The mallet, he said, was a powerful condensing instrument. He had used it in the earliest years of his practice, but abandoned it out of respect for *authority*. He had again taken it up out of respect for his own judgment and *conscience*. He had successfully filled frail teeth by using it that he could not so regulate the force as to fill without it.

In preparing cavities, when the enamel is under cut, or not strong, it was important to level, or counter sink the margins. And in filling with cylinders, he said, adhesive foil should not be used, as it would choke the cavity.

In reply to questions, he stated that if the canal was thoroughly clean, he did not care whether it was filled or not; and that his habit was to leave a pledget of cotton, saturated with creosote, high up in the canal and file over it.

It was now moved and carried that the final adjournment of this Convention take place at 12 M., to-morrow, and that it hold its next annual meeting at SARATOGA SPRINGS, the first Tuesday of August, 1860.

Adjourned till 9 A. M., to-morrow.

FOURTH DAY, AUGUST 5TH.

Dr. S. P. MILLER exhibited an electric apparatus for use in the extraction of teeth.

V. *Mechanical Dentistry.*

Dr. FRANKLIN expressed a high degree of dissatisfaction at the shortness of the time allotted to the consideration of this subject. It had not had a fair hearing in any meeting of the Convention, but had been crowded into a brief space, near the close of the meeting.

Dr. PEABODY, through Dr. Chase, forwarded a paper detailing his method of making casts. This paper we have not a copy of, but as the same modes, substantially, have been described before, we hope no loss is sustained by its omission.

Dr. DUNN made some extended remarks on the porcelain base (Loomis' patent), and

Dr. LOOMIS read a paper on the same subject, but as they were *entirely* eulogistic, and not in any way explanatory, they will excuse their omission from our report.

Dr. FULLER, of Peekskill, described his mode of antagonizing teeth. He finishes the upper plate, then adjusts the lower bicuspids, to correspond with them, solders these on, and then arranges the remaining lower teeth by them.

Dr. BAILEY thought the porcelain work was likely, in some cases, to make the lip too prominent.

Dr. DUNN thought the gum would be made thinner by using it, than the gum and plate combined, as in ordinary work.

Dr. ALLEN eulogized the "continuous gum" work, and then went on to state that, in mending it, he uses a more fusible body than the piece is composed of. In antagonizing, it was important to observe the law, that the superstructure must not overhang the base; it was, therefore, desirable to have the inner cusps meet first. He insisted on the importanee of artistic taste, in arranging artificial teeth, and maintained that weight was no objection to an artificial denture.

Dr. GOLDEY said that though weight might be no objection, yet patients do object to it. He thought that great weight in the lower set causes absorption of the ridge.

Dr. ROBERTS said that it was maintained, yesterday, that pressure on deciduous fangs does not cause absorption. Was it any more reasonable that pressure causes absorption of the alveolar ridge.

Dr. BUTLER thought that the process absorbs faster when a temporary plate is worn; and he accounted for it on the principle of pressure.

Dr. Roberts thought the absorption was less rapid, in such cases.

Dr. Chase inquired of Dr. Allen if he had not difficulty in continuous gum work, from the contraction of the gum body, in baking; and if he did not break the teeth, in re-adjusting the plate to the cast.

Dr. Allen replied that he forces the plate to the cast, after baking, and did not recollect of a case in which the teeth broke.

Dr. Chase described his method of trimming the plaster model so as to allow for contraction.

Dr. Franklin remarked that it ought to be generally known that plaster expands for five hours after it is mixed with water. Any one could try it by filling a glass bottle with it. He had seen the fit of a plate destroyed, by the expansion of the plaster, in making an antagonizing model. To obviate this, he would lay a fold of soft paper in the plate, before putting it in the plaster.

He recommended great care in melting zinc. It should be taken from the fire before the entire mass is melted, and stirred with a pine stick to prevent crystalization till it is ready to pour; and it should be used, for melting, only four or five times.

In baking continuous gum work, he said, if the teeth were set on their cutting edges, the plate would be less likely to spring.

The discussion of the sixth subject in the report was omitted.

VII. *Professional Intercourse.*

Dr. Wilson read a paper which will be published.

Dr. Taylor also read a paper, which will be published.

The time for final adjournment was extended till 1 P. M.; and "Miscellaneous Business" was taken up.

On motion of Dr. Fuller, the thanks of the Convention were tendered to the retiring officers, and also to Dr. Rogers, for his efficiency as a committee, in making the arrangements for this meeting.

On motion of Dr. Fuller, Dr. L. W. Rogers, the acting president, was appointed a Committee to make like arrangements, for the next meeting, at Saratoga.

Dr. TAFT exhibited a maxillary tumor, which had been removed from the mouth of a patient of Dr. Tefft. Time did not admit an examination of the tumor, or a history of the case.

Dr. DUNN explained his method of enlarging the models, so as to atone for shrinkage in baking the porcelain work. The description was illustrated with specimens, without which it would be difficult to give a clear description of the process.

Dr. PERINE exhibited Dr. Levett's patent atmospheric pressure plate. For a description of this, and any information concerning it, address Dr. Levett, New York.

Dr. FRANKLIN exhibited a cup for taking lower impressions in partial sets.

Dr. FORBES read the following paper from a Chemist of St. Louis, which explains itself:

Dr. ISAIAH FORBES.

*Dear Sir:*—With great pleasure I shall give you an account of the experiments we have been trying in order to find the composition of a new cement for the teeth. You remember that early last Spring I found a notice in some German scientific periodical of an excellent cement which had been sent from Paris to the dentists of Munich. Mr. Feichtinger of the latter place had analyzed it, and found it to be the oxychloride of zinc, discovered and described by Sorel, with some addition to improve its durability. We prepared it according to his communications, but could not succeed in getting a cement as we wanted it, and had, therefore, to try our own skill. After some fruitless experiments, our efforts were rewarded with success, and what is more than that, with your approval. It may be that we, perhaps, can improve the article, and I shall pay to it again some attention as soon as my new laboratory will be finished. The present cement consists of two materials, which, mixed together, produce a firm plastic mass.

No. 1. The powder consists of—

| | |
|---|---|
| Freshly calcined oxide of zinc . . . . . | 9 parts. |
| Finely powdered borax . . . . . . . | 1 part. |
| Finely powdered silex . . . . . . . | 2 parts. |

All mixed well together.

No. 2. The liquid is merely a concentrated solution of chloride of zinc in the smallest possible quantity of silicate of soda or soluble glass.

Respectfully, your most obedient servant,

ENNO SANDER.

On motion adjourned.

☞ The reporter intended to give a list, with description, of all the apparatus, instruments and appliances which were on exhibition at the Convention, but he found it to be wholly impracticable to do so. Some of these will be noticed in the *Register* hereafter.

